# Pharmacokinetics and tissue distribution of Ramulus Mori (Sangzhi) alkaloids in rats and its effects on liver enzyme activity

**DOI:** 10.3389/fphar.2023.1136772

**Published:** 2023-02-17

**Authors:** Zhihua Liu, Yu Feng, Hang Zhao, Jinping Hu, Yanmin Chen, Dongdong Liu, Hongliang Wang, Xiangyang Zhu, Hongzhen Yang, Zhufang Shen, Xuejun Xia, Jun Ye, Yuling Liu

**Affiliations:** ^1^ Beijing Wehand-Bio Pharmaceutical Co, Ltd., Beijing, China; ^2^ State Key Laboratory of Bioactive Substance and Function of Natural Medicines, Institute of Materia Medica, Chinese Academy of Medical Sciences & Peking Union Medical College, Beijing, China; ^3^ Beijing Key Laboratory of Drug Delivery Technology and Novel Formulation, Institute of Materia Medica, Chinese Academy of Medical Sciences & Peking Union Medical College, Beijing, China

**Keywords:** ramulus mori (Sangzhi) alkaloids, LC-MS/MS, pharmacokinetics, tissue distribution, metabolite

## Abstract

Ramulus Mori (Sangzhi) alkaloids (SZ-A) derived from twigs of mulberry (*Morus alba* L., genus *Morus* in the Moraceae family) was approved by the National Medical Products Administration in 2020 for the treatment of type 2 diabetes mellitus. In addition to excellent hypoglycemic effect, increasing evidence has confirmed that SZ-A exerts multiple pharmacological effects, such as protecting pancreatic *ß*-cell function, stimulating adiponectin expression, and alleviating hepatic steatosis. Importantly, a specific distribution of SZ-A in target tissues following oral absorption into the blood is essential for the induction of multiple pharmacological effects. However, there is a lack of studies thoroughly exploring the pharmacokinetic profiles and tissue distribution of SZ-A following oral absorption into the blood, particularly dose-linear pharmacokinetics and target tissue distribution associated with glycolipid metabolic diseases. In the present study, we systematically investigated the pharmacokinetics and tissue distribution of SZ-A and its metabolites in human and rat liver microsomes, and rat plasma, as well as its effects on the activity of hepatic cytochrome P450 enzymes (CYP450s). The results revealed that SZ-A was rapidly absorbed into the blood, exhibited linear pharmacokinetic characteristics in the dose range of 25–200 mg/kg, and was broadly distributed in glycolipid metabolism-related tissues. The highest SZ-A concentrations were observed in the kidney, liver, and aortic vessels, followed by the brown and subcutaneous adipose tissues, and the heart, spleen, lung, muscle, pancreas, and brain. Except for the trace oxidation products produced by fagomine, other phase I or phase II metabolites were not detected. SZ-A had no inhibitory or activating effects on major CYP450s. Conclusively, SZ-A is rapidly and widely distributed in target tissues, with good metabolic stability and a low risk of triggering drug-drug interactions. This study provides a framework for deciphering the material basis of the multiple pharmacological functions of SZ-A, its rational clinical use, and the expansion of its indications.

## 1 Introduction

Type 2 diabetes mellitus (T2DM) is one of the most important chronic metabolic diseases that threaten human health worldwide (Zheng et al., 2018). The pathogenesis of T2DM is complex and involves changes in various target tissues and organ functions, including impaired pancreatic islet *ß*-cell function and abnormal *a*-cell secretion (Campbell and Newgard, 2021); insulin resistance; lipid accumulation in the liver, muscle, adipose, and other tissues (Ke et al., 2022; SantaCruz-Calvo et al., 2022); impaired enteropancreatic axis function; intestinal flora imbalance; renal glucose reabsorption dysfunction (Mudaliar et al., 2015); brain tissue nerve transmitter secretion disorders (Scherer et al., 2021; Mirzadeh et al., 2022); and macrophage inflammation. Among the therapeutic agents currently used for the treatment of T2DM, glucagon-like peptide-1 agonists and dipeptidyl peptidase-4 inhibitors primarily exert their therapeutic effects by acting on the pancreatic islets, brain, and gastrointestinal tract, whereas metformin and thiazolidinediones function by reducing insulin resistance in the liver, muscles, and fat. Sodium-dependent glucose transporters two act on the kidney to inhibit glucose reabsorption, whereas *a*-glucosidase inhibitors primarily act on *a*-glucosidase in the gastrointestinal tract to delay or inhibit glucose uptake (Tahrani et al., 2016). Owing to the complex pathogenesis of T2DM, current clinical treatments recommend a multiplet strategy: therapeutic agents should not only reduce blood glucose parameters considerably but should also have substantial benefits in terms of cardiovascular safety, reduced glycemicity and prevention of T2DM complications (Perreault et al., 2021). Consequently, there is an urgent need for the continuous development of therapeutic agents with multiple pharmacological effects that contribute to the comprehensive treatment and clinical benefits of T2DM.

Ramulus Mori (Sangzhi) alkaloids (SZ-A) are a group of polyhydroxy alkaloids extracted and isolated from the traditional Chinese medicine mulberry twig (*Morus alba* L.), accounting for more than 50% of the total mulberry twig extract, which is majorly composed of 1-deoxynojirimycin (DNJ); fagomine (FA); and 1,4-dideoxy-1,4-imino-D-arabinitol (DAB) (Jin et al., 2022). Among these three components, DNJ contains the highest alkaloid content, accounting for >50% of the total alkaloids. The sum of the three main components accounts for more than 80% of the total alkaloid content. As a natural hypoglycemic drug, SZ-A tablets were approved by the China National Medical Products Administration (NMPA) in 2020 for the treatment of T2DM (approval number Z20200002). The results of a randomized double-blind phase III clinical trial with the chemical drug acarbose tablets (Glucobay^®^) as the positive control (No. CTR20140034) revealed that SZ-A could effectively reduce glycated hemoglobin levels in patients and the hypoglycemic efficacy of SZ-A was comparable to that of acarbose during 24 weeks of treatment (Qu et al., 2021). Importantly, the incidence of drug-related adverse events and gastrointestinal adverse reactions induced by SZ-A was significantly lower than that induced by acarbose, indicating that SZ-A exhibited superior safety.

*α*-glucosidase inhibition was one of the hypoglycemic mechanisms discovered earlier for SZ-A. SZ-A is more selective for glycosidase species than acarbose and primarily acts on disaccharidase, with almost no amylase inhibition (Hiele et al., 1992; Liu et al., 2019). This pharmacological feature is conducive to faster and better control of postprandial blood glucose, while greatly reducing adverse reactions such as flatulence and exhaust in East Asian patients consuming carbohydrates as their staple food (Nojima et al., 1998; Liu et al., 2021). In addition to selectively inhibiting intestinal *a*-glucosidase, an in-depth study of the mechanism underlying the effects of SZ-A found that exerts multiple *in vitro* and *in vivo* pharmacological effects on the regulation of pancreatic islet *ß*-cell function, insulin resistance, lipid metabolism, intestinal flora, and inflammation (Cao et al., 2021; Liu et al., 2021; Chen et al., 2022; Lei et al., 2022; Sun et al., 2022). In a previous study, the therapeutic effects and mechanism of action of SZ-A on lipid metabolism were explored in high-fat diet (HFD)-induced obesity and non-alcoholic fatty liver disease (NAFLD) mice. We found that orally administered SZ-A ameliorated HFD-induced weight gain and significantly stimulated adiponectin expression and secretion in the adipose tissue. Additionally, SZ-A markedly reduced hepatic steatosis and regulated lipid metabolism and oxidative stress in the liver. To further investigate the target tissues that mediate the protective effect of SZ-A against obesity and NAFLD, SZ-A was intraperitoneally administered to HFD-induced mice. Notably, HFD-induced obesity, hepatic steatosis, oxidative stress, infiammation, and fibrosis in mice were also ameliorated by intraperitoneal administration of SZ-A. These results indicated that intestinal *a*-glucosidase was not the only target of SZ-A, and SZ-A might also be absorbed into the blood following oral administration and widely distributed in T2DM-related target tissues, such as the pancreatic islets, liver, muscle, and adipose tissue (Schwartz et al., 2017), and then act directly on these tissues to exert multiple pharmacological effects. This pharmacological feature of SZ-A differs remarkably from that of the traditional glucosidase inhibitor acarbose, which primarily functions in the gastrointestinal tract and is barely absorbed into the blood (Krentz and Bailey, 2005).

An important pre-requisite for the multiple pharmacological effects of SZ-A on T2DM-related tissues is the specific distribution of SZ-A in the target tissues following oral absorption into the blood. Although multiple pharmacological effects of SZ-A have been confirmed in previous studies, the knowledge regarding its distribution in T2DM-related tissues (including muscle, adipose tissue, etc.,) is still unclear. Preliminary pharmacokinetic results following SZ-A administration in rats showed that the absolute bioavailability of the three active components of SZ-A (DNJ, FA, and DAB) was >70%, implying widespread tissue distribution (Yang et al., 2017). However, there is a lack of studies thoroughly exploring the pharmacokinetic profiles and tissue distribution with regard to its oral absorption into the blood especially dose-linear pharmacokinetics and target tissue distribution associated with glycolipid metabolic diseases. Additionally, it remains unclear whether SZ-A is metabolized by the liver and affects the activity of liver microsomal isozymes. The safety of a drug is closely related to its metabolism in the liver, its effects on the activity of liver enzymes, and drug-drug interactions.

**SCHEME 1:**
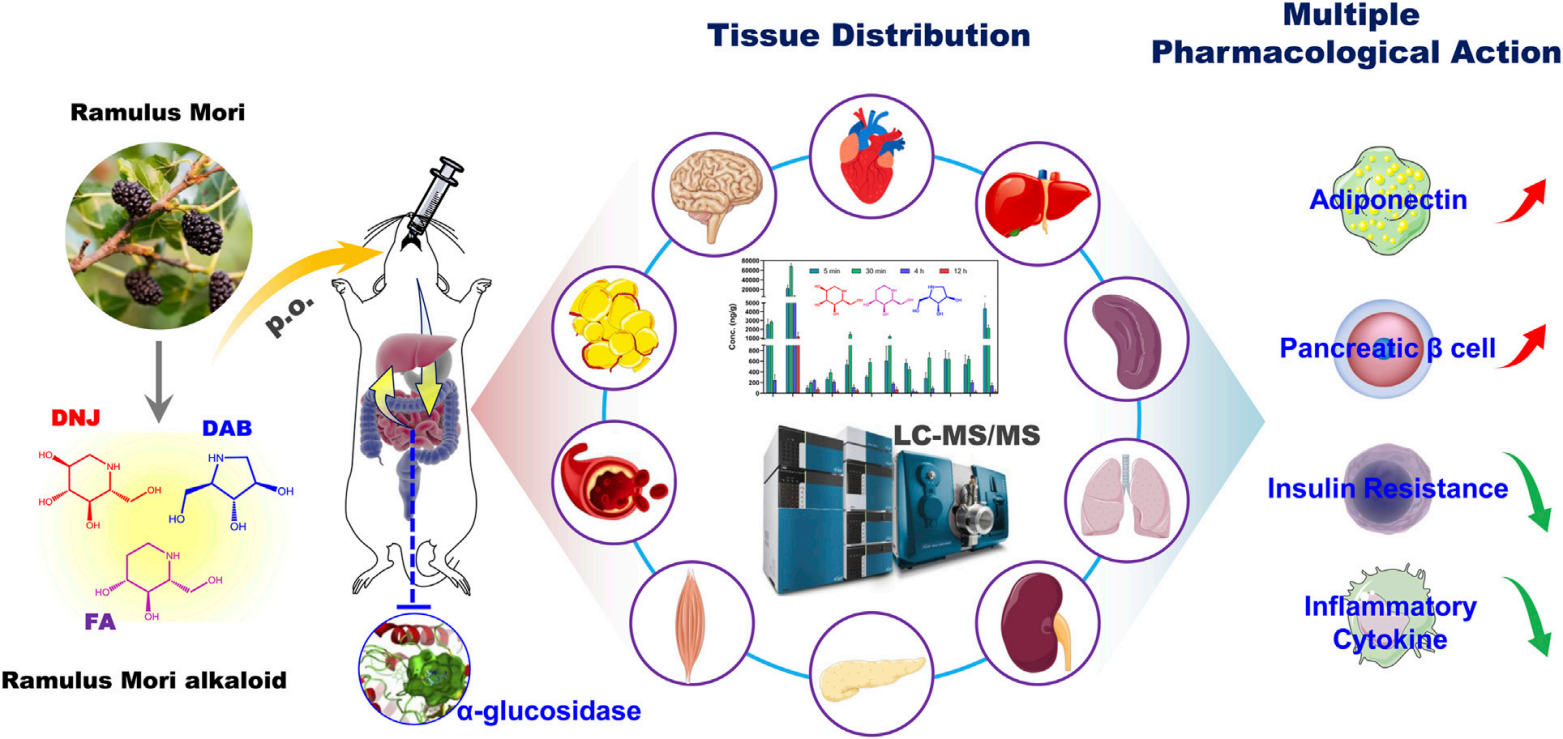
Schematic illustration of the pharmacokinetics and tissue distribution of SZ-A in rats after oral administration. SZ-A is rapidly absorbed into the blood and widely distributed in the type 2 diabetes mellitus (T2DM)-related tissues after oral administration, which lays the material basis for its multiple pharmacological effects *in vivo* such as stimulating the secretion of adiponectin, protecting pancreatic *ß*-cell function, improving insulin resistance, and reducing the secretion of inflammatory cytokines.

To fully understand the tissue distribution characteristics of SZ-A following oral administration, the distribution of the three active components of SZ-A (DNJ, FA, and DAB) in 12 tissues, including the heart, liver, spleen, lung, kidney, aortic blood vessels, brown adipose tissue, subcutaneous adipose tissue, abdominal adipose tissue, muscle, pancreas, and brain, was determined in mice and rats (Scheme 1). Metabolites in human/rat liver microsomes (HLMs/RLMs) or rat plasma were identified to evaluate whether SZ-A was metabolized by the liver. Furthermore, the effect of SZ-A on cytochrome P450 enzymes (CYP450s) activity was investigated to evaluate the possibility of SZ-A-induced drug-drug interactions. A better understanding of the tissue distribution characteristics of SZ-A is beneficial to further explore its multiple pharmacological effects and mechanisms and provide a material foundation for the subsequent expansion of new therapeutic indications.

## 2 Materials and methods

### 2.1 Materials

DNJ (purity >99.0%) and SZ-A extract (Lot No.: J202004007, containing 36.88% of DNJ, 8.78% of FA, and 5.83% of DAB; Lot No.: J202108007, containing 36.52% of DNJ, 9.60% of FA, and 7.62% of DAB) were provided by Beijing Wehand-bio Pharmaceutical Co. Ltd. (Beijing, China). The multiple reaction monitoring (MRM) chromatogram of SZ-A is shown in Supplementary Figure S1. Miglitol was obtained from TCI Shanghai Chemical Industrial Development Co., Ltd. (Shanghai, China). FA (purity >98.0%) was purchased from MedChemExpress (Monmouth Junction, NJ, United States ). DAB (purity >98.0%) was purchased from Sigma-Aldrich (St. Louis, MO, United States ). Human liver microsomes (HLMs) were purchased from Reid Liver Disease Research (Shanghai, China). Glucose-6-phosphate, oxidized coenzyme H (β-NADP), glucose-6-phosphate dehydrogenase, midazolam, phenacetin, dextromethorphan, mephenytoin, chlorzoxazone, diclofenac sodium, 1-Hydroxy-Midazolam, 4-hydroxy-mephenytoin, acetaminophen, 4-Hydroxy-Diclofenac Sodium, demethyldextromethorphan, 6-Hydroxy-Chlorzoxazone, furaphylline, sulfafenpyrazole, quinidine, ketoconazole, and sodium diethyldithiacarbamate were purchased from Sigma-Aldrich (St. Louis, MO, United States ). All other organic reagents were of analytical grade and purchased from Sinopharm Chemical Reagent (Shanghai, China).

### 2.2 Animals

For the pharmacokinetic study, Sprague-Dawley rats (SD, 180–220 g, both male and female) were purchased from Speifu (Beijing) Biotechnology Co., Ltd. (Beijing, China). Animal experiments were approved by the Ethics Committee of Kangtai Medical Laboratory Service Hebei Co., Ltd. (Hebei, China) (No. MDL 2022-01-17-1).

For the tissue distribution study, male SD rats (180–200 g) and male ICR mice (20–25 g) were purchased from Speifu (Beijing) Biotechnology Co., Ltd. (Beijing, China). The ambient temperature and humidity in the laboratory were maintained at approximately 22°C and 50%, respectively. Animal experiments were approved by the Ethics Committee of Zhongsheng Beidong (Beijing) Technology Development Co., Ltd. (Beijing, China) (No. 20200039YFE-3R and 20200084YZE-3R).

### 2.3 Pharmacokinetic study

Twenty-four SD rats were fasted for 12 h (free access to water) and randomly classified into three groups (*n* = 8; half male and female) that received different doses of SZ-A solution (25, 50, and 200 mg/kg) *via* oral administration. After completion of dose administration, blood samples (0.3 mL) were collected through an indwelling catheter from the jugular vein at predetermined time points (5, 15, 30, and 45 min, 1, 2, 4, 8, and 24 h). The blood was centrifuged at 5,000 rpm for 10 min to separate the plasma. All plasma samples were stored frozen at −80°C until liquid chromatography coupled to tandem mass spectrometry (LC-MS/MS) analysis (AB SCIEX Triple Quad™ 4,500 mass spectrometer, Applied Biosystems Inc., United States).

### 2.4 Tissue distribution

Twenty SD rats were randomly classified into four groups corresponding to four-time points (5 and 30 min, 4 and 12 h) (*n* = 20; 5 animals per time point) that received SZ-A solution (40 mg/kg) *via* oral administration. After the completion of dose administration, plasma and tissue samples (derived from the heart, liver, spleen, lung, kidney, aortic blood vessels, brown adipose tissue, subcutaneous adipose tissue, abdominal adipose tissue, muscle (thigh skeletal muscle), pancreas, and brain) were collected at predetermined time points. Twenty-eight ICR mice were randomly classified into four groups (*n* = 7 per group) corresponding to four-time points (5 and 30 min, 4 and 12 h) that received SZ-A solution (20 mg/kg) *via* oral administration. After the completion of dose administration, tissue samples (derived from the heart, liver, spleen, lung, kidney, aortic blood vessels, brown adipose tissue, abdominal adipose tissue, muscle (thigh skeletal muscle), pancreas, and brain) were collected at predetermined time points.

Collected tissue samples were washed with normal saline. The surface moisture was removed by blotting gently with filter paper, and the weight was recorded. After adding an appropriate amount of normal saline, these tissue samples were ground into homogenates using a cryogenic grinder (JX-CL, Shanghai Jingxin, Shanghai, China). The obtained homogenates were stored at −80°C until LC-MS/MS analysis.

### 2.5 Inhibition of CYP450s

The experiments were classified into six groups (*n* = 3): control (no inhibitor), positive inhibitor, SZ-A (final concentration, 8.899 μg/mL; equivalent to 20 μM DNJ), DNJ (final concentration, 20 μM), FA (final concentration, 5 μM), and DAB (final concentration, 8 μM). Different inhibitors were added to the HLMs/RLMs and incubated with the probe substrates of each subtype.

The liver microsomal incubation system comprised human or rat liver microsomal protein (0.5 mg/mL), nicotinamide adenine dinucleotide phosphate (NADPH) generation system, Tris-HCl buffer (50 mM, pH 7.4), substrate, and inhibitor, with a total reaction volume of 200 μL. The substrate concentrations and incubation times for CYP1A2, 2D6/2D2, 2C9/2C6, 2C19/2C11, 3A4/3A2, and 2E1 were phenacetins, 50 μM/30 min; dextromethorphan, 5 μM/10 min; diclofenac sodium, 20 μM/10 min; mephenytoin, 40 μM/30 min; midazolam, 5 μM/5 min; and chlorzoxazone, 80 μM/20 min, respectively. The concentrations of each isoenzyme-positive inhibitor were 10 μM of furafylline (50 μM in the rat group), 2 μM quinidine (25 μM in the rat group), 5 μM sulfaphenazole (50 μM in the rat group), 5 μM of diclofenac pyridine (25 μM in the rat group), 1 μM ketoconazole, and 80 μM sodium diethyldithiacarbamate (100 μM in the rat group). After the reaction was completed, 400 μL of the internal standard propranolol (final concentration, 200 ng/mL) in ice-cold acetonitrile was added to terminate the reaction, and the sample was mixed well and centrifuged twice at 14,000 rpm for 5 min. Five microliters of the supernatant were collected for LC-MS/MS analysis (UPLC-Q-Extractive Orbitrap MS; Thermo Fisher Scientific, Waltham, MA, United States) to determine the metabolite content of the probe substrate.

### 2.6 Identification of metabolites

The metabolites in the 6 h plasma samples from the pharmacokinetic study and the HLMs/RLMs reaction system in the SZ-A group from the CYP450s inhibition study were identified using Q-Extractive high-resolution MS.

### 2.7 LC-MS/MS analysis

The concentration of SZ-A in plasma and tissue was determined using LC-MS/MS analysis. Two hundred microlitres of miglitol solution (internal standard, 100 ng/mL, dissolved in acetonitrile-methanol solution (5:2, v/v, 0.1% formic acid)) and 10 µL methanol-water (4:1, v/v) were added to 50 µL of plasma or tissue homogenate, mixed by vortexing for 2 min, and centrifuged at 14,000 rpm for 10 min. One hundred microliters of the supernatant were mixed with 100 µL acetonitrile-water (3:1, v/v, 0.1% formic acid), vortexed for 1 min, and centrifuged at 14,000 rpm for 5 min. Five microliters of the supernatant were injected into the LC-MS/MS system. The analysis was performed using an LC-MS/MS system comprising an Exion LC AC HPLC system and a Triple Quad 4500 MS system. The three active components of SZ-A (DNJ, FA, and DAB) and miglitol were separated by using a chromatographic column (XBridge™ Amide, 3.5 µm, 4.6 × 150 mm) at 35°C. Mobile phases A and B contained 0.1% ammonia in water and acetonitrile, respectively. A gradient elution program was used with a flow rate of 0.5, 0–4 min for 65% mobile phase B, 4–7 min for 43% mobile phase B, and 7–13 min for 65% mobile phase B. The injection volume was 5 µL. The mass spectrometer was equipped with an electrospray ionization source and scanned in the multiple reaction monitoring mode and positive ion mode. The main parameters were as follows: curtain gas (CUR): 35.0 psi, collision gas (CAD): 9, ion spray voltage (IS): 5500V, temperature (TEM): 500°C, ion source gas1 (GS1): 55.0 psi, ion source gas2 (GS2): 50.0 psi, entrance potential (EP): 10.0 eV; collision cell exit potential (CXP): 6.0 eV. The ion reaction pairs used for monitoring were as follows: DNJ, m/z 164.3→69.0 (DP 70ev, CE 27ev); FA, m/z 148.2→112.1 (DP 56ev, CE 18ev); DAB, m/z 134.3→68.1 (DP 55ev, CE 25ev); and miglitol, m/z m/z 208.3→146.1 (DP 70ev, CE 27ev).

For the CYP450s inhibition study, LC-MS/MS detection conditions were as follows: chromatographic column, Zorbax C18 column (3.5 μm, 2.1 × 100 mm); mobile phase, acetonitrile (0.1% formic acid) and water (0.1% formic acid); gradient elution; flow rate, 0.2 mL/min; ionization mode, CYP2E1 was negative; MS conditions, acetaminophen (m/z: 152→110), 4-hydroxymephenytoin (m/z:235→150), desmethyldextromethorphan (m/z: 258→157), 4-hydroxydiclofenac (m/z:312→230), and 1-hydroxymidazolam (m/z: 342→203) in positive ion mode and 6-hydroxychlorzoxazone (m/z:184→120) in negative ion mode. The scan parameters were as follows: resolution, 70,000; AGC target, 200,000; and maximum IT, 100 ms (enzyme effect).

For the metabolite identification study, rat plasma or liver microsomes were precipitated with acetonitrile 1:4 (v:v), vortexed for 60 s, and centrifuged twice at 14,000 rpm for 5 min. Furthermore, 5 μL of the supernatant was collected for LC-MS/MS analysis. The LC-MS/MS conditions were as follows: chromatographic column (XBridge™ Amide, 3.5 µm, 4.6 × 150 mm); mobile phase, water (0.1% ammonia) and acetonitrile, other conditions are the same as those in CYP450s inhibition study, with the following scan parameters: resolution, 70,000; AGC target, 3,000,000; maximum IT, 200 ms; and scan range, 50–600 m/z.

### 2.8 Pharmacokinetic parameters analysis

Primary pharmacokinetic parameters, including C_max_, T_max_, area under the plasma concentration-time curve (AUC), t_1/2_, clearance rate, and the apparent volume of distribution, were calculated using DAS 3.2.8 (Bio Guider Co., Shanghai, China).

### 2.9 Statistical analysis

Statistical analysis was performed using a Student’s t-test for two groups or one-way analysis of variance (ANOVA) for more than two groups using GraphPad Prism version 7.00 for Windows (GraphPad Software, La Jolla, CA, United States ). Differences were considered statistically significant if the *p*-value was less than 0.05.

## 3 Results and discussion

### 3.1 Method validation of LC-MS/MS

**FIGURE 1:**
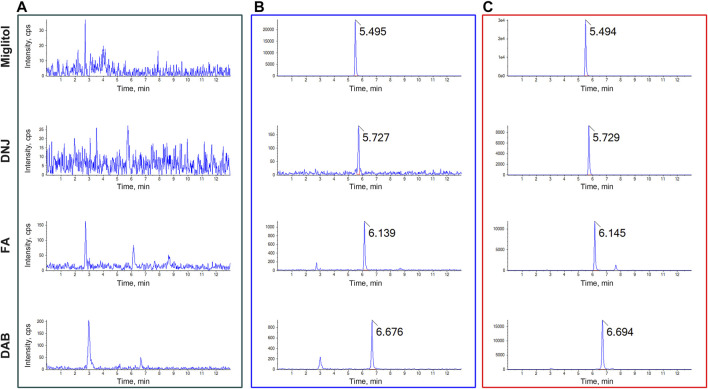
Typical multiple reaction monitoring (MRM) chromatograms for **(A)** blank plasma **(B)** blank plasma spiked with DNJ, FA, DAB, and miglitol, and **(C)** plasma samples after oral administration of SZ-A. The peak at 5.495, 5.727, 6.139, and 6.676 min were miglitol, DNJ, FA, and DAB, respectively.

Systematic validation of all LC-MS/MS analytical methods included in this study was performed to meet the acceptance criteria recommended by the Food and Drug Administration (FDA) guidelines for Industry Bioanalytical Method Validation. No obvious endogenous interference was found at the retention times of DNJ, FA, DAB, and miglitol (internal standard) in the blank plasma or plasma samples. Representative chromatograms of blank rat plasma, blank plasma sampleS spiked with DNJ, FA, DAB, and miglitol, as well as the plasma sample from the SZ-A-administered rats, are shown in Figure 1.

The calibration curves for DNJ, FA, and DAB in rat plasma were linear over a concentration range of 50–5,000 ng/mL for DNJ, 25–2,500 ng/mL for FA, and 25–2,500 ng/mL for DAB. The corresponding linear regression equation with a 1/x^2^ weighting factor was y = 1.85020e-4x + 0.00191 (*r* = 0.9997) for DNJ, *y* = 4.41781e-4 x + 4.01643e-4 (*r* = 0.9996) for FA, and *y* = 0.00123 x + 0.00188 (*r* = 0.9990) for DAB, where y represents the ratio of the analyte peak area to the miglitol area, and x is the analyte concentration. The other linear regression equations for the calibration curves of DNJ, FA, and DAB in rat tissues are presented in Table S1. The correlation coefficients (r) were >0.99 for all calibration curves. The average accuracy of the calibration standards for all three analytes was between 85.47%–113.33%, with a relative standard deviation (RSD) of 1.43%–13.57%. Additionally, the results of the intra- and inter-batch accuracy and precision, matrix effect, extraction recovery, and stability met the acceptance criteria recommended by the FDA guidelines, demonstrating that the developed LC-MS/MS analytical methods can be used to accurately determine the concentrations of DNJ, FA, and DAB in plasma and tissues.

### 3.2 Pharmacokinetic study of SZ-A in rats

**FIGURE 2:**
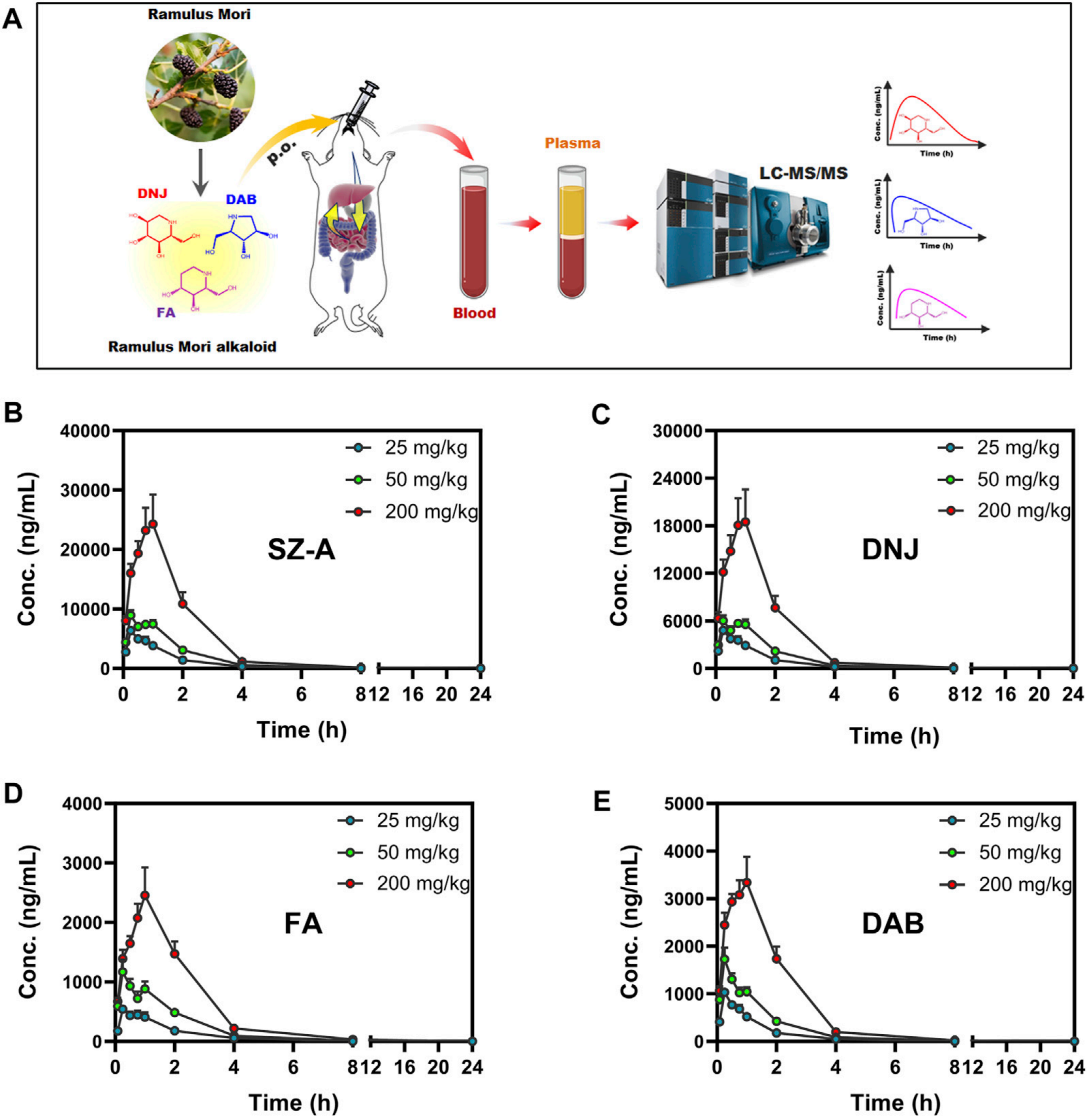
Experimental scheme of plasma concentration determination after oral administration of SZ-A (25, 50, and 200 mg/kg) in rats **(A)**. Mean plasma concentration-time profiles of SZ-A **(B)**, DNJ **(C)**, FA **(D)**, and DAB **(E)** in rats following oral administration of SZ-A. Each value represents the mean ±standard error of mean (SEM) (*n* = 8).

The plasma concentration-time curves and main pharmacokinetic parameters after administration of different doses of SZ-A (25, 50, and 200 mg/kg) in SD rats are shown in Figure 2 and Supplementary Table S2. As shown in Figure 2, the three active components of SZ-A, DNJ, FA, and DAB, were detected in plasma 5 min after oral administration and reached their maximum plasma concentrations (T_max_) within 0.88 h, indicating the rapid absorption of the three alkaloids from the gastrointestinal tract. The plasma drug concentration increased with the dose with similar T_max_ regardless of the dose groups. For the oral administration dose of 50 mg/kg SZ-A, the half-life (t_1/2_) of DNJ, FA, and DAB in plasma were 1.07 ± 0.04, 1.19 ± 0.03, and 1.20 ± 0.03 h, respectively. The volume of distribution (Vz) of DNJ, FA, and DAB in plasma was 4.47 ± 1.30, 6.88 ± 1.85, and 4.70 ± 0.65 L/kg, respectively. The short half-life and large volume of distribution demonstrated that the three alkaloids were rapidly eliminated from the blood and transported to tissues or organs. Additionally, the oral absolute bioavailability of DNJ, FA, and DAB was 72.41%, 77.50%, and 78.23%, respectively, after oral administration of SZ-A in rats (Yang et al., 2017). Taken together, the short half-life, large volume of distribution, and high oral absolute bioavailability of DNJ, FA, and DAB laid the foundation for further study of their dynamic tissue distribution *in vivo*.

**FIGURE 3:**
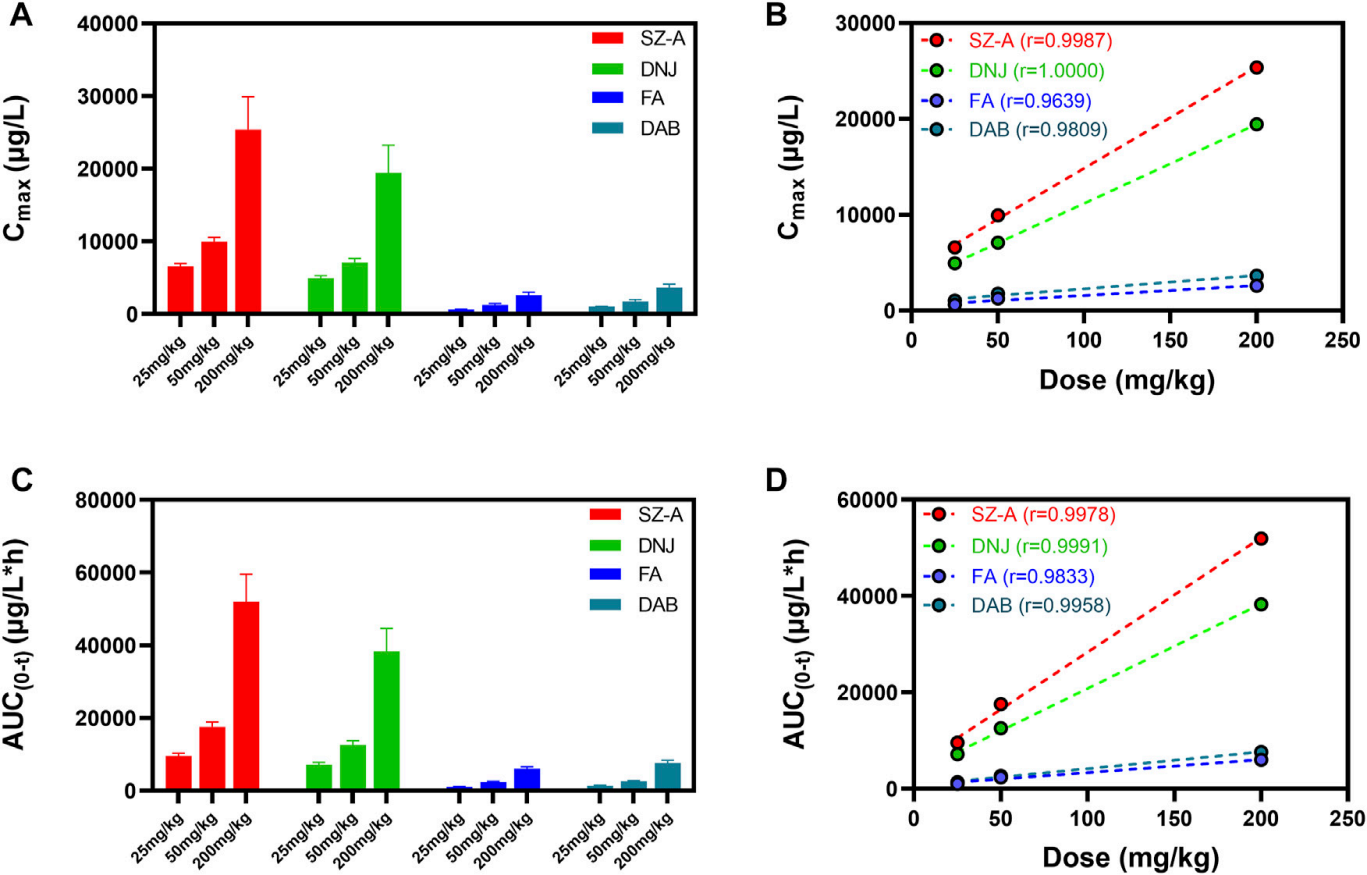
The linear relation between C_max_ and the SZ-A dose **(A,B)** and between AUC and the SZ-A dose **(C,D)**. Each value represents the mean ± standard error of mean (SEM) (*n* = 8).

As shown in Figure 3, an obvious linear relationship was observed between C_max_ and the SZ-A dose and between AUC_(0-t)_ and SZ-A dose. All the correlation coefficient values (*r* ^2^) for AUC_(0-t)_ *versus* dose and C_max_ *versus* dose were above 0.9, demonstrating that C_max_ and AUC_(0-t)_ have strong positive correlations with the SZ-A dose. Additionally, for DNJ, no significant differences were observed in t_1/2_ and CLz between the three doses. These results indicated that the SZ-A exhibited a linear pharmacokinetic profile when administered orally at a dose range of 25–200 mg/kg.

These results are consistent with our previous experimental findings, such as rapid absorption of the three alkaloids from the gastrointestinal tract, fast elimination from the blood, and rapid distribution into tissues (Yang et al., 2017). However, what we found in a preliminary study of DNJ, FA, and DAB exhibited non-linear pharmacokinetics following oral administration of SZ-A at the investigated dosage in rats (20–500 mg/kg), which is contrary to our current findings (Yang et al., 2017). According to the above results, SZ-A presented a linear pharmacokinetic profile in the range of 25–200 mg/kg and non-linear pharmacokinetics at higher doses of 200–500 mg/kg following oral administration in rats.

### 3.3 Tissue distribution of SZ-A in rats

In a previous study, we found that SZ-A could exert multiple pharmacological effects on T2DM-related target tissues, including adipose tissue and the liver, by oral or intraperitoneal administration (Chen et al., 2022). For example, SZ-A ameliorated HFD-induced weight gain and significantly stimulated adiponectin expression and secretion in adipose tissue (Sun et al., 2022). Additionally, SZ-A alleviates hepatic steatosis and regulates lipid metabolism and oxidative stress in the liver (Chen et al., 2022). These results suggested that intestinal *a*-glucosidase was not the only target of SZ-A and that SZ-A might be widely distributed in T2DM-related target tissues after oral administration, followed by direct action on these tissues to exert multiple pharmacological effects. To fully investigate the tissue distribution characteristics of SZ-A after oral administration, the concentrations of the three active components of SZ-A in plasma and twelve tissues including all T2DM-related target tissues, were determined.

**FIGURE 4:**
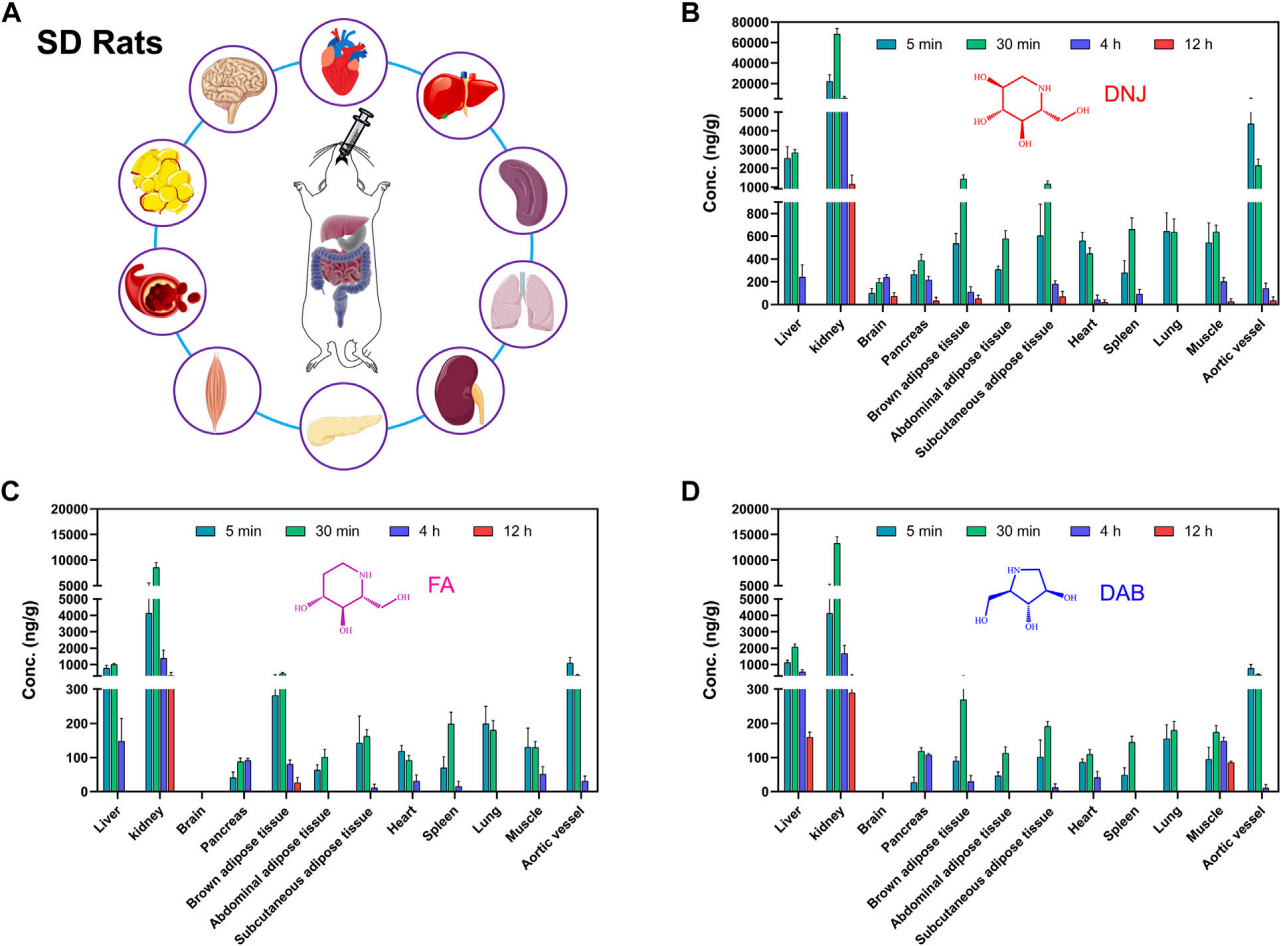
Experimental scheme of tissue concentration determination after oral administration of SZ-A in rats **(A)**. The concentration of the three active components of SZ-A, including DNJ **(B)**, FA **(C)**, and DAB **(D)**, in tissues, including heart, liver, spleen, lung, kidney, aortic blood vessel, brown adipose tissue, subcutaneous adipose tissue, abdominal adipose tissue, muscle, pancreas, and brain, after a single oral administration of SZ-A (40 mg/kg) in SD rats. Each value represents the mean ± standard error of mean (SEM) (*n* = 5).

The mean concentrations of the three active components of SZ-A (DNJ, FA, and DAB) in plasma and tissues, including the heart, liver, spleen, lung, kidney, aortic blood vessels, brown adipose tissue, subcutaneous adipose tissue, abdominal adipose tissue, muscle, pancreas, and brain, after a single oral administration of SZ-A (40 mg/kg) in SD rats are shown in Supplementary Figure S2 and Figure 4. As shown in Figure 4, the distribution patterns of DNJ, FA, and DAB over time in each tissue at 5, 30 min, 4 h, and 12 h were consistent. The three alkaloids were rapidly and widely distributed in various tissues after the oral administration of SZ-A, and the highest concentrations of DNJ, FA, and DAB were detected in the kidney, followed by the liver, aortic vessels, and brown adipose tissue. These tissues are most closely associated with weight loss and glucose lowering (Cypess et al., 2014; Baskin et al., 2015; Kajimura et al., 2015). The distribution rates of the three alkaloids in the same tissue were approximately the same. Of all the tissues, distribution was fastest in the heart and aortic vessels. The concentration of DNJ peaked 5 min after administration in the heart and aortic vessels and 30 min after administration in other tissues, and the peak time in the brain was the longest (4 h). FA reached the maximum drug concentration in the heart, muscle, lung, and aortic vessels at 5 min and other tissues at 30 min. DAB was most rapidly distributed in the aortic vessel within 5 min and the other tissues at 30 min. After 12 h of oral administration, all three alkaloids were rapidly eliminated from most of the tissues. Supplementary Table S3 summarizes the main pharmacokinetic parameters. The results showed that the half-life (t_1/2_) of SZ-A in the liver, kidney, pancreas, muscle, and brain was approximately 2 h, whereas that in other tissues was less than 1 h. The clearance rate (CLz) of SZ-A in the kidney was lower than 0.5 L/h/kg, while the clearance rate in abdominal adipose tissue, heart, spleen, and lung was higher than 10 L/h/kg. These results demonstrate that the three main alkaloids of SZ-A can be rapidly distributed in various tissues after oral administration, which may be due to its high oral bioavailability.

The study of tissue distribution is an important part of the pharmacokinetic study. The *in vivo* distribution characteristics are the basis for evaluating the pharmacology and toxicity of drugs. Typically, multiple pharmacological effects of drugs are closely related to their wide tissue distribution. After oral administration, SZ-A underwent rapid distribution into tissues within the time course examined, and no long-term accumulation of the three alkaloids DNJ, FA, and DAB in tissues were observed. The major tissue distributions of DNJ, FA, and DAB were similar. The highest tissue concentrations of all three alkaloids were observed in the kidney, followed by the liver, aortic vessels, and brown adipose tissue.

Studies have shown that DNJ can normalize renal function in diabetic rats, suggesting renal protection against diabetes (Huang et al., 2014). The distribution of SZ-A in the kidney supports its direct renoprotective effect. Coronary atherosclerotic heart disease is a typical macrovascular complication of diabetes (DeFronzo et al., 2015), and studies have found that DNJ has pleiotropic effects on the development of atherosclerosis by inhibiting glucose-stimulated vascular smooth muscle cell migration through activating AMP-activated Protein Kinase (AMPK)/RhoB and downregulating focal adhesion kinase (FAK) (Chan et al., 2013). The high concentration of SZ-A distributed in the aortic vessels has positive significance for the treatment of atherosclerosis complicated by diabetes. The distribution of SZ-A in the liver is vital for alleviating hepatic steatosis and regulating fatty acid metabolism, lipid accumulation, and oxidative stress, thereby alleviating non-alcoholic fatty liver disease and reducing body weight in high-fat diet-induced obese mice (Chen et al., 2022). DNJ participates in the glucose transport system by directly regulating the protein expression of glycolytic and gluconeogenic enzymes, which inhibit intestinal glucose absorption and accelerate hepatic glucose metabolism (Li et al., 2013). Notably, SZ-A also has a high drug distribution in brown adipose tissue, abdominal adipose tissue, and subcutaneous adipose tissue, which is of great significance in reducing insulin resistance, regulating adipokine secretion, and controlling body weight. Our previous study showed that SZ-A could improve lipid metabolism and inhibit weight gain in HFD-induced obese mice by inhibiting fatty acid synthase and increasing lipolytic enzyme expression to inhibit fat accumulation. Additionally, SZ-A has been found to have a beneficial effect on obesity-induced chronic inflammation in adipose tissue (Sun et al., 2022). All the current studies have shown that SZ-A plays a role in adipose tissue, and our discovery of the distribution of the main components of SZ-A in adipose tissue provides a foundation for further research on its mechanism in regulating adipose tissue.

This study also found that SZ-A has a certain drug distribution in T2DM-related tissues, such as the pancreas, muscle, spleen, lung, and heart, and can pass through the blood-brain barrier and become rapidly distributed in the brain tissue. Lipid sensing and insulin signaling in the brain independently trigger negative feedback systems that can effectively reduce glucose production and food intake to restore metabolic homeostasis in T2DM and obesity (Yue and Lam, 2012). The function of the gut-brain axis is often attributed to gastrointestinal hormones, and gut gluconeogenesis plays an important role in the regulation of energy homeostasis in the brain. An increasing number of studies have shown that hyperglycemia is associated with cognitive decline and that defective insulin signaling may increase the risk of Alzheimer’s disease (Strachan et al., 2011; Lupaescu et al., 2022). These studies suggest that SZ-A has the possible potential to treat brain aging and neuroinflammation.

### 3.4 Tissue distribution of SZ-A in mice

**FIGURE 5:**
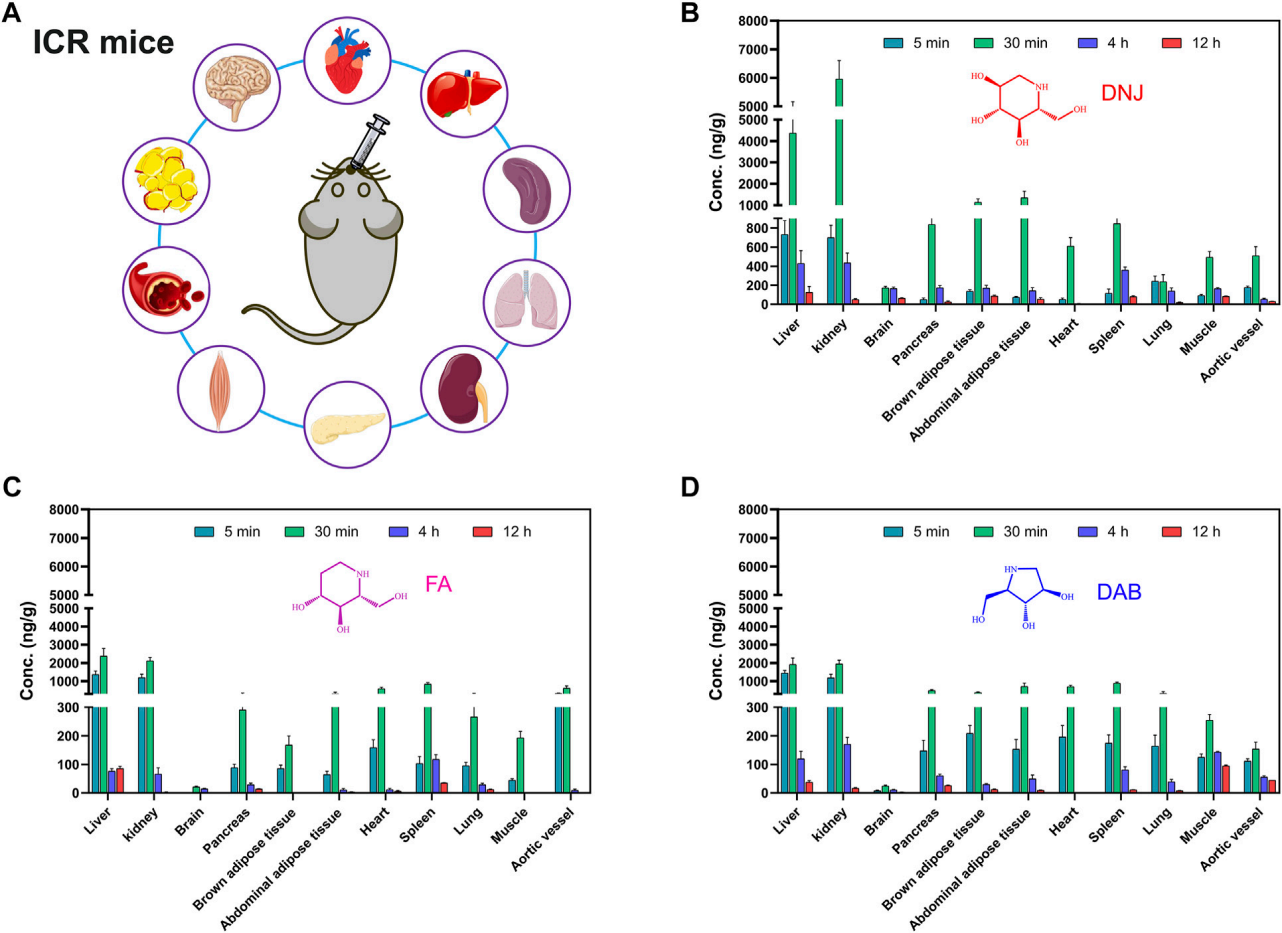
Experimental scheme of tissue concentration determination after oral administration of SZ-A in mice **(A)**. The concentration of the three active components of SZ-A, including DNJ **(B)**, FA **(C)**, and DAB **(D)**, in tissues, including heart, liver, spleen, lung, kidney, aortic blood vessels, brown adipose tissue, abdominal adipose tissue, muscle, pancreas, and brain, after a single oral administration of SZ-A (20 mg/kg) in ICR mice. Each value represents the mean ± standard error of mean (SEM) (*n* = 7).

The concentrations of the three active components of SZ-A (DNJ, FA, and DAB) in the heart, liver, spleen, lung, kidney, aortic blood vessels, brown adipose tissue, abdominal adipose tissue, muscle, pancreas, and brain after a single oral administration of SZ-A (20 mg/kg) in ICR mice are shown in Figure 5. As shown in Figure 5, the distribution patterns of DNJ, FA, and DAB over time in each tissue at 5, 30 min, 4 h, and 12 h were consistent. The three alkaloids were rapidly and widely distributed in various tissues after oral administration of SZ-A, and the highest concentrations of DNJ, FA, and DAB were detected in the kidney, followed by the liver. In contrast to the tissue distribution characteristics of rats, the drug distribution to the tissues of mice was slower than that of rats because the drug concentration in each tissue of mice at 5 min was much lower than that at 30 min. The distribution of DNJ and DAB in aortic vessels was lower than that in other tissues, and the distribution of DAB in the pancreas, brown adipose tissue, abdominal adipose tissue, heart, and spleen was similar but higher than that in aortic vessels. The distribution of FA in the pancreas, abdominal adipose tissue, heart, spleen, and aortic vessels was similar. These results indicate that SZ-A can be rapidly distributed to the tissues of mice, and the tissue distribution characteristics are similar to those of rats, demonstrating that the distribution characteristics of SZ-A in different species of animals show little difference.

### 3.5 Inhibitory effects of CYP450s on HLMs/RLMs

Liver microsome assays used positive inhibitors such as quinidine, ketoconazole, sulfaphenazole, ticlopidine, furafylline, and sodium diethyldithiacarbamate, which have inhibitory effects on CYP450s in HLMs. The metabolic inhibition rates of probe substrates were 84.19%, 90.44%, 84.04%, 68.46%, 71.84%, and 53.09%, for CYP2D6, 3A4, 2C9, 2C19, 1A2, and 2E1 from HLMs, respectively, and 56.56%, 76.37%, 88.00%, 65.17%, 66.33%, and 46.91% for these enzymes from RLMs, respectively. These results also indicate that the incubation system used in this experiment is sensitive and reliable.

**FIGURE 6:**
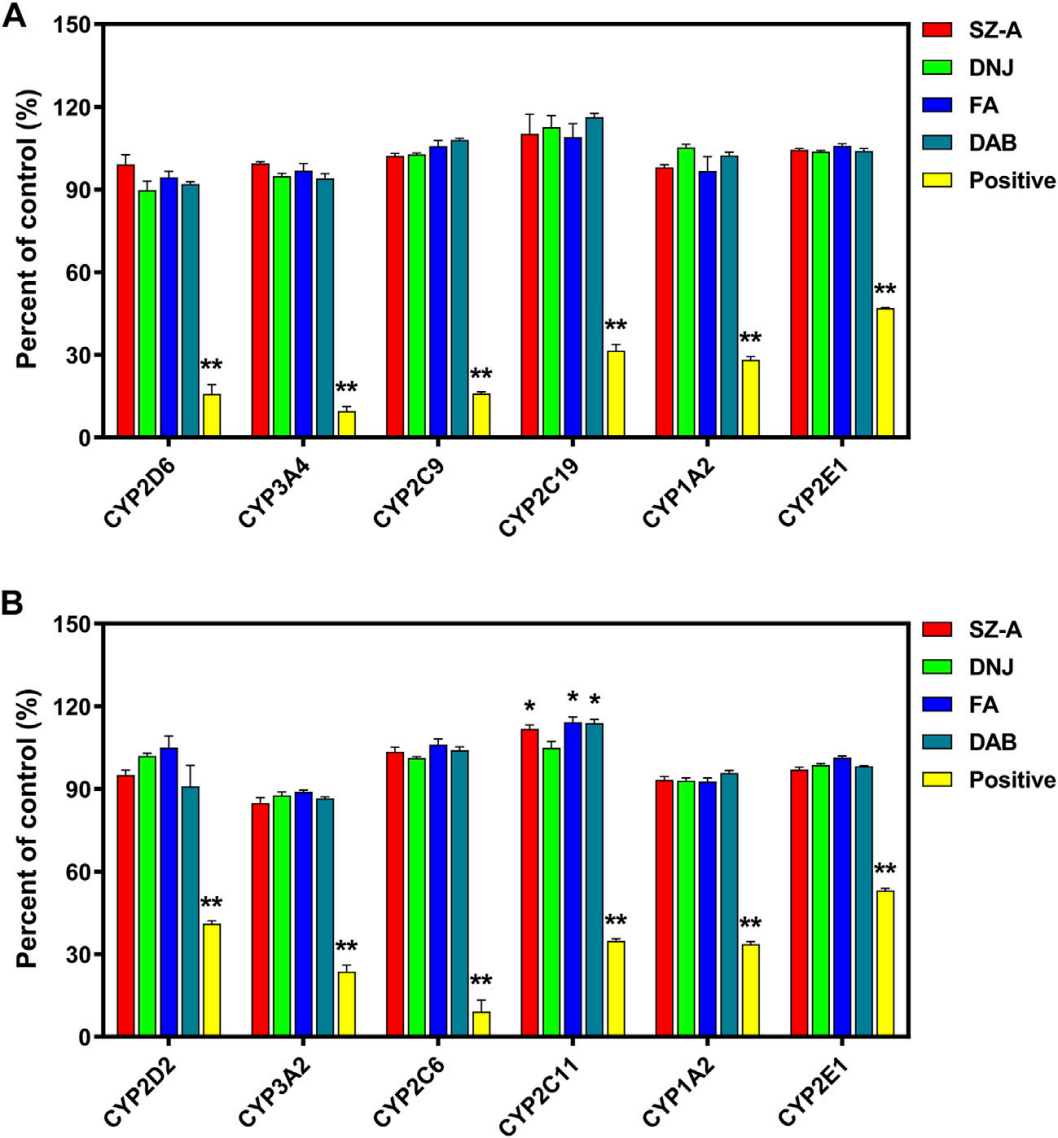
The effect of SZ-A on the activity of CYP450s in human liver microsomes (HLMs, **(A)**) and rat liver microsomes (RLMs, **(B)**). Each value represents the mean ± standard error of mean (SEM) (*n* = 3). *p < 0.05, **p < 0.01 compared with the control group.

The effect of SZ-A on the activity of CYP450s in HLMs and RLMs is shown in Figure 6. SZ-A (final concentration 8.899 μg/mL, equivalent to DNJ at 20 μM), DNJ (final concentration, 20 μM), FA (final concentration, 5 μM), and DAB (final concentration, 8 μM) had no significant effect on the major CYP450s in HLMs, either inhibition or activation. They had a mild activation effect on rat CYP2C11 but no obvious effect on other isoenzymes.

CYP450s play a leading role in all kinds of enzymes involved in drug metabolism (Zhao et al., 2021). The inhibition or induction of its activity is the main reason for metabolic drug interactions. Enzyme inhibition is more clinically significant than enzyme induction, accounting for approximately 70% of metabolic interactions. No obvious inhibition or activation of CYP2D6, 3A4, 2C9, 2C19, 1A2, and 2E1 isoenzyme activities were found in the SZ-A and human liver microsome incubation system. SZ-A has a mild activating effect on rat CYP2C11 but has no significant effect on other isoenzymes. CYP2C11 is a rat protein corresponding to human CYP2C9, whereas SZ-A has no significant effect on human liver microsome CYP2C9, which might be due to species differences. This study showed that SZ-A is less likely to exhibit drug-drug interactions based on CYP450s in clinical medication, which also provides an important reference for further research on the concomitant administration of SZ-A, expanding its clinical applications.

### 3.6 Identification of metabolites

**FIGURE 7:**
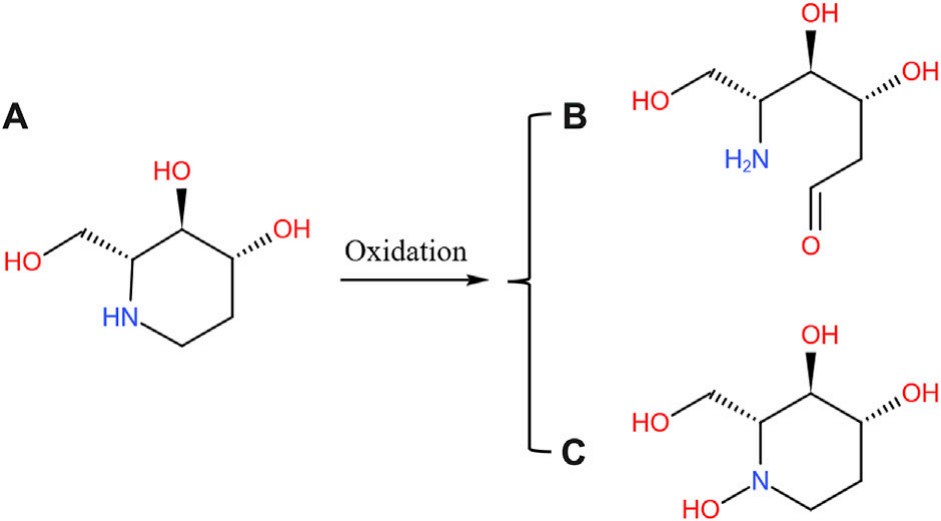
Possible oxidation pathway of FA **(A)** and its metabolites **(B)** and **(C)**. The metabolites of SZ-A were identified from the plasma samples of rats after oral administration of SZ-A (40 mg/kg) and the human liver microsomes (HLMs) and rat liver microsomes (RLMs) samples in the enzyme inhibition study.

With the help of Q Extractive high-resolution MS, a full scan was performed on SZ-A and the three main active components in HLMs/RLMs samples incubated at body temperature and in rat plasma. The metabolites were analyzed using chromatographic retention time and accurate molecular weight. Except for trace amounts of FA oxidation products, no other related metabolites were found in HLMs/RLMs and rat plasma. The quasi-molecular ion peak of FA was (M + H)^+^ m/z 148.09682, and its molecular formula was C_6_H_13_NO_3_. Its metabolite quasi-molecular ion peak was (M + H)^+^ m/z 164.09173 and its molecular formula was C_6_H_13_NO_4_. Compared to the parent drug FA, the quasi-molecular ion of the FA oxidation metabolite increased by one oxygen atom, and the relative molecular mass increased by 16 u. The position of nitrogen could be a possible active site, and thus, it was speculated that there might be two types of FA oxidation metabolite structures: one that opens the ring at the nitrogen position to form COHCH_2_(CHOH)_2_CH(CH_2_OH)NH_2_ and the other that is not ring-opened and oxidized on the nitrogen atom (Figure 7). The lack of structural confirmation of oxidation metabolites of FA is a limitation of the present study. The main components of SZ-A, DNJ, FA, and DAB exist mainly in the parent form, whether in the liver microsome incubation system or after oral absorption into the blood, indicating that SZ-A has excellent metabolic stability.

Drug metabolism in the body is closely related to its efficacy and safety. There was no detectable phase I and phase II metabolites of DNJ or DAB found in HLMs/RLMs and rat plasma reaction system, whereas only trace oxidation products were detected for FA. The position of nitrogen in the FA molecular structure is a possible activation site, and it is speculated that there might be two types of product structures after the oxidation reaction at this site; however, neither has an obvious toxic structure. According to another report, prototypes DNJ, FA, and DAB reached material conservation within 24 h after the oral administration of SZ-A, suggesting that they were mainly excreted in their original forms (Yang et al., 2017). Kiyotaka et al. did not detect any DNJ metabolites in the plasma of rats after oral gavage of DNJ (Nakagawa et al., 2007). Furthermore, DAB is mainly excreted unchanged in rats (Mackay et al., 2003). These studies suggest that SZ-A is not metabolized by the liver after being absorbed into the blood through the gastrointestinal tract, and has good metabolic stability.

## 4 Conclusion

SZ-A was rapidly absorbed into the blood following oral administration and showed linear pharmacokinetics in a dose range of 25–200 mg/kg in rats. The distribution of SZ-A in the pancreas, liver, and adipose tissues lays the foundation for its role in regulating insulin secretion and glucose and lipid metabolism. The wide distribution of SZ-A in the kidney, aorta, muscle, and other tissues suggests that SZ-A can also act on multiple target organs, and its multiple pharmacological effects deserve further study. SZ-A was primarily excreted in its original form and was not metabolized by the liver, which had no significant impact on the activity of liver isoenzymes, suggesting that SZ-A caused a low risk of potentially harmful drug-drug interactions.

## Data Availability

The original contributions presented in the study are included in the article/Supplementary Material, further inquiries can be directed to the corresponding authors.
