# Supplementary material for: Pharmacokinetics and tissue distribution of Ramulus Mori (Sangzhi) alkaloids in rats and its effects on liver enzyme activity
Source: Front Pharmacol. 2023 Feb 17;14:1136772. doi: 10.3389/fphar.2023.1136772 (PMC9981942; doi:10.3389/fphar.2023.1136772)
Supplement: Supplementary file 1 [file DataSheet1.DOCX]

Supplementary Material

Article Title

First Author*, Co-Author, Co-Author

*** Correspondence:** Corresponding Author: email@uni.edu

# Supplementary Figures and Tables


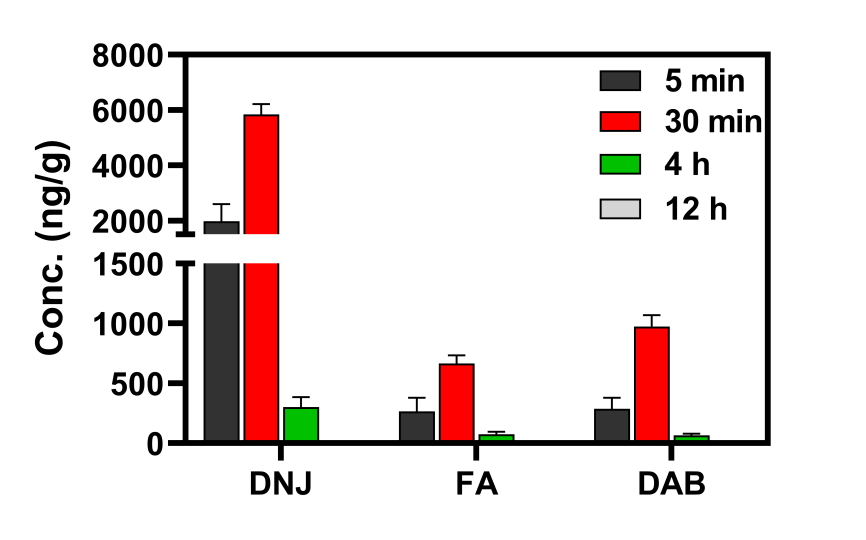

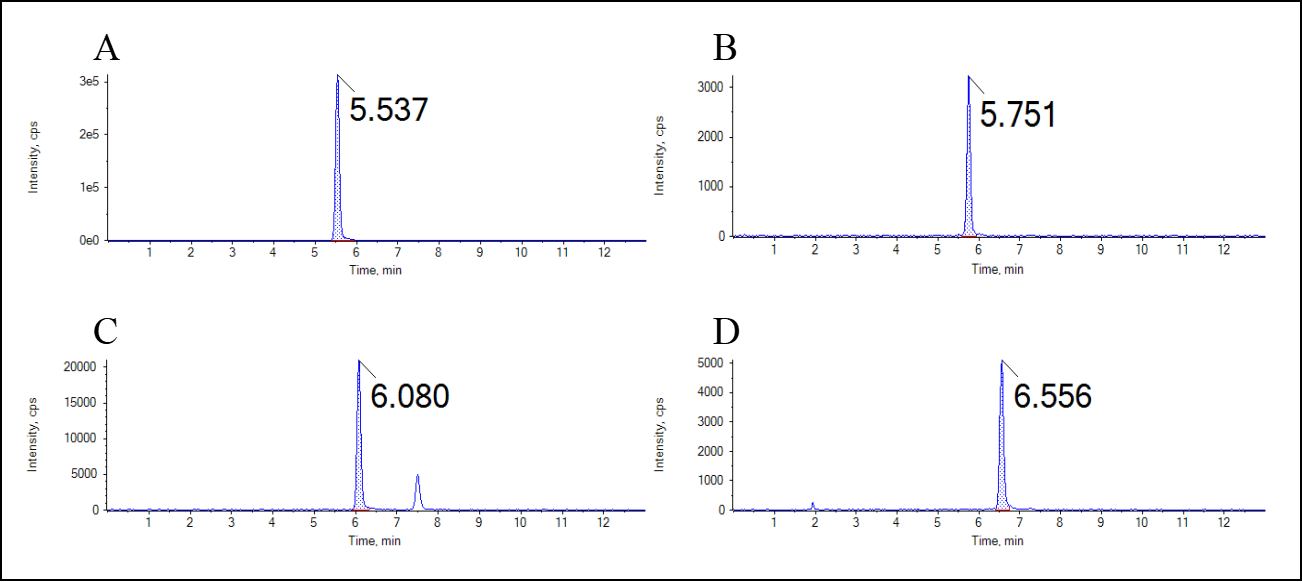


**Figure S1.** The MRM chromatogram of SZ-A, miglitol (A), DNJ (B), FA (C), and DAB (D).

**Figure S2.** Mean plasma concentration of DNJ, FA, and DAB in rats following oral administration of SZ-A (40 mg/kg). Each value represents the mean ± SEM (n = 5).

## Table S1. The linear regression equation of the calibration curves of DNJ, FA, and DAB in tissues of rats

| **Biological Samples** | **Chemicals** | **Regression Equation** | **Correlation** | **Linear Range** |
| --- | --- | --- | --- | --- |
|  |  |  | **Coefficient (r2)** | **(ng/mL or (ng/g)** |
| Plasma | DNJ | y = 1.34465e-4 x + 0.00259 | 0.99805 | 50-5000 |
|  | FA | y = 8.95990e-4 x + 0.00263 | 0.99936 | 25-2500 |
|  | DAB | y = 8.53429e-4 x + 8.50191e-4 | 0.99973 | 25-2500 |
| Liver | DNJ | y = 5.24888e-4 x + 3.21406e-4 | 0.99822 | 50-5000 |
|  | FA | y = 0.00217 x + -0.00708 | 0.99916 | 25-2500 |
|  | DAB | y = 0.00252 x + -0.01484 | 0.99901 | 25-2500 |
| Kidney | DNJ | y = 2.27518e-4 x + 0.00231 | 0.99587 | 50-5000 |
|  | FA | y = 0.00113 x + -0.00389 | 0.99929 | 25-2500 |
|  | DAB | y = 0.00142 x + -0.00533 | 0.99905 | 25-2500 |
| Pancreas | DNJ | y = 2.32681e-4 x + 0.00188 | 0.99999 | 25-2500 |
|  | FA | y = 0.00101 x + 0.00271 | 0.99868 | 12.5-1250 |
|  | DAB | y = 0.00119 x + 2.93198e-4 | 0.99754 | 12.5-1250 |
| Brain | DNJ | y = 1.56550e-4 x + 0.00359 | 0.99969 | 25-2500 |
|  | FA | y = 7.41119e-4 x + 0.00170 | 0.99950 | 12.5-1250 |
|  | DAB | y = 7.18065e-4 x + 6.00748e-4 | 0.99959 | 12.5-1250 |
| Muscle | DNJ | y = 1.93129e-4 x + 0.00420 | 0.99951 | 25-2500 |
|  | FA | y = 6.60302e-4 x + 0.00217 | 0.99925 | 12.5-1250 |
|  | DAB | y = 0.00115 x + 8.37497e-4 | 0.99927 | 12.5-1250 |
| Brown adipose tissue | DNJ | y = 1.32030e-4 x + 0.00258 | 0.99918 | 25-2500 |
|  | FA | y = 8.33199e-5 x + -2.23109e-4 | 0.99947 | 12.5-1250 |
|  | DAB | y = 7.22466e-4 x + 0.00118 | 0.99928 | 12.5-1250 |
| Subcutaneous adipose tissue | DNJ | y = 1.66443e-4 x + 0.00316 | 0.99975 | 25-2500 |
|  | FA | y = 8.24439e-4 x + 0.00201 | 0.99975 | 12.5-1250 |
|  | DAB | y = 7.88501e-4 x + 6.85235e-4 | 0.99968 | 12.5-1250 |
| Abdominal adipose tissue | DNJ | y = 1.65574e-4 x + 0.00270 | 0.99926 | 25-2500 |
|  | FA | y = 8.17775e-4 x + 7.89668e-4 | 0.99939 | 12.5-1250 |
|  | DAB | y = 7.79353e-4 x + 3.65568e-4 | 0.99922 | 12.5-1250 |
| Heart | DNJ | y = 1.56429e-4 x + 0.00514 | 0.99946 | 25-2500 |
|  | FA | y = 9.07281e-4 x + 0.00152 | 0.99895 | 12.5-1250 |
|  | DAB | y = 8.66467e-4 x + 7.92122e-4 | 0.99986 | 12.5-1250 |
| Spleen | DNJ | y = 1.61098e-4 x + 0.00442 | 0.99738 | 25-2500 |
|  | FA | y = 7.85030e-4 x + 0.00112 | 0.99902 | 12.5-1250 |
|  | DAB | y = 6.60010e-4 x + 4.52368e-4 | 0.99952 | 12.5-1250 |
| Lung | DNJ | y = 1.50754e-4 x + 0.00381 | 0.99367 | 25-2500 |
|  | FA | y = 7.50032e-4 x + -4.04183e-4 | 0.99689 | 12.5-1250 |
|  | DAB | y = 6.63787e-4 x + -0.00101 | 0.99502 | 12.5-1250 |
| Aortic blood vessels | DNJ | y = 1.71337e-4 x + 0.00262 | 0.99669 | 25-2500 |
|  | FA | y = 9.01836e-4 x + 0.00118 | 0.99858 | 12.5-1250 |
|  | DAB | y = 8.20183e-4 x + 3.31140e-4 | 0.99965 | 12.5-1250 |

## Table S2. The main pharmacokinetic parameters after different oral administration doses of SZ-A (25, 50, and 200 mg/kg) in SD rats (n=8)

|  | **Parameter** | **25 mg/kg** | **50 mg/kg** | **200 mg/kg** |
| --- | --- | --- | --- | --- |
| DNJ | t1/2 (h) | 1.02±0.27 | 1.07±0.04 | 3.25±4.20 |
|  | Tmax (h) | 0.41±0.23 | 0.63±0.33 | 0.78±0.25 |
|  | Vz (L/kg) | 3.65±1.45 | 4.47±1.30 | 17.77±20.83 |
|  | CLz (L/h/kg) | 2.50±0.67 | 2.88±0.76 | 4.47±2.65 |
|  | Cmax (μg/L) | 4948.84±978.38 | 7075.56±1640.33 | 19443.69±10777.16 |
|  | AUC(0-t) (μg/L*h) | 7160.04±1646.75 | 12566.48±3471.45 | 38297.51±18008.69 |
|  | AUC(0-∞) (μg/L*h) | 7160.04±1646.75 | 12566.48±3471.45 | 38545.47±18022.70 |
|  | MRT(0-t) (h) | 1.27±0.20 | 1.51±0.42 | 1.79±0.30 |
|  | MRT(0-∞) (h) | 1.27±0.20 | 1.51±0.42 | 2.02±0.59 |
|  | VRT(0-t) (h^2) | 1.15±0.57 | 2.02±1.71 | 6.42±4.65 |
|  | VRT(0-∞) (h^2) | 1.15±0.57 | 2.02±1.71 | 16.10±21.66 |
| FA | t1/2 (h) | 1.16±0.31 | 1.19±0.03 | 1.08±0.04 |
|  | Tmax (h) | 0.50±0.30 | 0.56±0.37 | 0.88±0.27 |
|  | Vz (L/kg) | 7.62±2.89 | 6.88±1.85 | 9.94±2.86 |
|  | CLz (L/h/kg) | 4.71±1.46 | 4.00±1.04 | 6.31±1.60 |
|  | Cmax (μg/L) | 616.20±168.45 | 1254.51±505.82 | 2583.98±1247.62 |
|  | AUC(0-t) (μg/L*h) | 1039.80±355.70 | 2372.67±634.04 | 6011.96±1653.29 |
|  | AUC(0-∞) (μg/L*h) | 1039.81±355.70 | 2372.67±634.04 | 6011.97±1653.29 |
|  | MRT(0-t) (h) | 1.55±0.23 | 1.67±0.61 | 1.97±0.22 |
|  | MRT(0-∞) (h) | 1.55±0.23 | 1.67±0.61 | 1.97±0.22 |
|  | VRT(0-t) (h^2) | 1.46±0.55 | 1.90±2.02 | 3.08±0.76 |
|  | VRT(0-∞) (h^2) | 1.46±0.55 | 1.91±2.02 | 3.08±0.76 |
| DAB | t1/2 (h) | 1.16±0.31 | 1.20±0.03 | 1.08±0.04 |
|  | Tmax (h) | 0.31±0.12 | 0.44±0.35 | 0.75±0.30 |
|  | Vz (L/kg) | 4.47±1.53 | 4.70±0.65 | 6.20±1.82 |
|  | CLz (L/h/kg) | 2.72±0.66 | 2.71±0.37 | 3.94±1.00 |
|  | Cmax (μg/L) | 1049.11±140.86 | 1765.09±632.47 | 3637.64±1369.97 |
|  | AUC(0-t) (μg/L*h) | 1363.80±298.92 | 2659.13±366.66 | 7631.55±2035.84 |
|  | AUC(0-∞) (μg/L*h) | 1363.80±298.92 | 2659.14±366.66 | 7631.55±2035.84 |
|  | MRT(0-t) (h) | 1.26±0.17 | 1.45±0.56 | 1.60±0.26 |
|  | MRT(0-∞) (h) | 1.26±0.17 | 1.45±0.56 | 1.60±0.26 |
|  | VRT(0-t) (h^2) | 1.25±0.51 | 1.83±2.07 | 1.92±0.81 |
|  | VRT(0-∞) (h^2) | 1.25±0.51 | 1.83±2.07 | 1.92±0.81 |
| SZ-A | t1/2 (h) | 0.99±0.26 | 1.04±0.03 | 2.51±2.84 |
|  | Tmax (h) | 0.34±0.19 | 0.53±0.39 | 0.72±0.31 |
|  | Vz (L/kg) | 3.88±1.48 | 4.41±0.84 | 14.79±15.31 |
|  | CLz (L/h/kg) | 2.75±0.71 | 2.93±0.50 | 4.53±2.15 |
|  | Cmax (μg/L) | 6575.30±1077.17 | 9939.33±1696.85 | 25370.35±12834.30 |
|  | AUC(0-t) (μg/L*h) | 9563.73±2074.50 | 17598.42±3655.03 | 51941.09±21503.19 |
|  | AUC(0-∞) (μg/L*h) | 9563.73±2074.50 | 17598.42±3655.03 | 52116.25±21516.97 |
|  | MRT(0-t) (h) | 1.29±0.19 | 1.52±0.46 | 1.77±0.22 |
|  | MRT(0-∞) (h) | 1.29±0.19 | 1.52±0.46 | 1.88±0.34 |
|  | VRT(0-t) (h^2) | 1.21±0.56 | 2.00±1.78 | 5.39±3.45 |
|  | VRT(0-∞) (h^2) | 1.21±0.56 | 2.00±1.78 | 9.13±9.31 |

## Table S3. The main pharmacokinetic parameters of tissues after oral administration of SZ-A (40 mg/kg) in SD rats (n=5)

|  | **Parameter** | **AUC_(0-t)_ (ug/L*h)** | **t_1/2_ (h)** | **T_max_ (h)** | **Vz (L/kg)** | **CLz (L/h/kg)** | **C_max_ (μg/L)** |
| --- | --- | --- | --- | --- | --- | --- | --- |
| DNJ | Plasma | 13651±1969 | 0.52±0.02 | 0.50±0.00 | 1.59±0.29 | 2.14±0.31 | 5833.77±842.92 |
|  | Liver | 7555±1845 | 0.55±0.01 | 0.42±0.19 | 3.15±0.69 | 3.97±0.90 | 3303.18±892.97 |
|  | Kidney | 174428±37115 | 2.46±0.51 | 0.50±0.00 | 0.60±0.20 | 0.17±0.03 | 68181.82±12390.67 |
|  | Pancreas | 2186±614 | 2.17±3.41 | 0.50±0.00 | 25.94±27.13 | 13.09±5.19 | 386.38±123.79 |
|  | Muscle | 2644±544 | 0.76±0.30 | 0.33±0.23 | 11.93±3.73 | 11.25±2.56 | 724.63±279.10 |
|  | Brown adipose tissue | 3748±995 | 0.44±0.22 | 0.50±0.00 | 5.34±3.66 | 8.17±2.54 | 1419.66±513.23 |
|  | Subcutaneous adipose tissue | 1207±304 | 0.63±0.00 | 0.50±0.00 | 22.40±4.23 | 24.68±4.67 | 577.20±162.96 |
|  | Abdominal adipose tissue | 3733±1010 | 1.70±2.25 | 0.42±0.19 | 15.37±15.15 | 8.08±3.00 | 1267.59±428.12 |
|  | Brain | 2059±562 | 8.04±7.17 | 2.52±2.04 | 77.20±61.64 | 10.48±6.04 | 242.27±50.26 |
|  | Heart | 1335±373 | 0.53±0.19 | 0.17±0.19 | 16.94±6.47 | 22.51±4.66 | 588.24±139.98 |
|  | Aortic blood vessels | 6230±1789 | 0.60±0.11 | 0.08±0.00 | 4.24±1.37 | 4.98±1.71 | 4364.01±2867.39 |
|  | Spleen | 1885±845 | 0.62±0.03 | 0.50±0.00 | 18.59±15.07 | 20.40±15.34 | 660.25±224.61 |
|  | Lung | 1222±501 | 0.47±0.24 | 0.22±0.24 | 15.44±3.37 | 27.06±13.42 | 805.60±23.51 |
| FA | Plasma | 1803±421 | 0.78±0.38 | 0.42±0.19 | 4.39±2.04 | 3.95±0.90 | 691.57±134.89 |
|  | Liver | 2987±1031 | 0.60±0.01 | 0.42±0.19 | 2.18±0.80 | 2.54±0.96 | 1129.44±187.00 |
|  | Kidney | 26953±8885 | 2.31±1.21 | 0.42±0.19 | 0.82±0.44 | 0.26±0.08 | 8968.60±1676.74 |
|  | Pancreas | 705±118 | 0.70±0.04 | 1.90±1.92 | 9.94±1.75 | 9.92±1.86 | 96.86±13.94 |
|  | Muscle | 577±298 | 0.68±0.04 | 0.33±0.23 | 15.19±9.50 | 15.29±9.05 | 175.04±91.23 |
|  | Brown adipose tissue | 1476±380 | 2.32±2.31 | 0.33±0.23 | 11.84±9.08 | 4.66±2.03 | 463.04±161.28 |
|  | Subcutaneous adipose tissue | 212±95 | 0.74±0.03 | 0.25±0.23 | 37.74±10.21 | 35.88±10.45 | 102.25±51.99 |
|  | Abdominal adipose tissue | 409±127 | 0.64±0.25 | 0.42±0.19 | 17.86±12.27 | 18.46±7.57 | 221.05±136.99 |
|  | Brain | / | / | / | / | / | / |
|  | Heart | 382±246 | 0.71±0.01 | 0.17±0.19 | 25.86±16.18 | 25.31±15.45 | 127.76±33.59 |
|  | Aortic blood vessels | 1059±367 | 0.45±0.24 | 0.08±0.00 | 4.48±2.36 | 7.17±2.74 | 1086.85±760.03 |
|  | Spleen | 491±264 | 0.70±0.08 | 0.50±0.00 | 19.17±13.60 | 18.04±10.52 | 197.76±77.73 |
|  | Lung | 367±136 | 0.52±0.26 | 0.22±0.24 | 13.48±4.29 | 20.59±8.23 | 248.16±5.46 |
| DAB | Plasma | 2357±349 | 0.57±0.02 | 0.50±0.00 | 1.61±0.24 | 1.95±0.26 | 973.37±215.23 |
|  | Liver | 8121±2152 | 3.82±0.74 | 0.50±0.00 | 2.95±1.03 | 0.54±0.17 | 2068.02±396.38 |
|  | Kidney | 37782±7168 | 2.75±0.51 | 0.50±0.00 | 0.48±0.14 | 0.12±0.02 | 13268.60±2886.19 |
|  | Pancreas | 854±90 | 0.67±0.04 | 1.90±1.92 | 5.16±0.58 | 5.35±0.61 | 121.89±16.95 |
|  | Muscle | 1553±212 | 15.48±11.67 | 1.12±1.62 | 26.15±7.39 | 1.43±0.45 | 187.72±38.37 |
|  | Brown adipose tissue | 716±259 | 0.68±0.01 | 0.50±0.00 | 7.13±3.26 | 7.22±3.25 | 268.60±82.78 |
|  | Subcutaneous adipose tissue | 232±75 | 0.74±0.03 | 0.50±0.00 | 22.15±5.03 | 20.90±5.22 | 112.41±41.52 |
|  | Abdominal adipose tissue | 460±146 | 0.73±0.06 | 0.42±0.19 | 11.39±4.52 | 10.66±3.43 | 211.76±58.19 |
|  | Brain | / | / | / | / | / | / |
|  | Heart | 469±213 | 0.71±0.01 | 1.20±1.57 | 12.63±7.93 | 12.29±7.57 | 114.39±24.24 |
|  | Aortic blood vessels | 962±354 | 0.46±0.24 | 0.08±0.00 | 3.59±2.62 | 5.35±2.21 | 755.64±535.60 |
|  | Spleen | 294±87 | 0.75±0.07 | 0.50±0.00 | 18.70±8.72 | 16.79±6.13 | 144.20±39.89 |
|  | Lung | 333±108 | 0.53±0.27 | 0.22±0.24 | 9.91±3.11 | 14.77±5.39 | 199.79±22.26 |
